# Single-cell genomics and spatial transcriptomics in islet transplantation for diabetes treatment: advancing towards personalized therapies

**DOI:** 10.3389/fimmu.2025.1554876

**Published:** 2025-02-20

**Authors:** Lisha Mou, Tony Bowei Wang, Yuxian Chen, Ziqi Luo, Xinyu Wang, Zuhui Pu

**Affiliations:** ^1^ Department of Endocrinology, Institute of Translational Medicine, Health Science Center, The First Affiliated Hospital of Shenzhen University, Shenzhen Second People’s Hospital, Shenzhen, Guangdong, China; ^2^ MetaLife Lab, Shenzhen Institute of Translational Medicine, Shenzhen, Guangdong, China; ^3^ Imaging Department, The First Affiliated Hospital of Shenzhen University, Shenzhen Second People’s Hospital, Shenzhen, Guangdong, China

**Keywords:** diabetes, ScRNA-seq, spatial transcriptomics, islet transplantation, islet graft, microenvironment

## Abstract

Diabetes mellitus (DM) is a global health crisis affecting millions, with islet transplantation emerging as a promising treatment strategy to restore insulin production. This review synthesizes the current research on single-cell and spatial transcriptomics in the context of islet transplantation, highlighting their potential to revolutionize DM management. Single-cell RNA sequencing, offers a detailed look into the diversity and functionality within islet grafts, identifying specific cell types and states that influence graft acceptance and function. Spatial transcriptomics complements this by mapping gene expression within the tissue’s spatial context, crucial for understanding the microenvironment surrounding transplanted islets and their interactions with host tissues. The integration of these technologies offers a comprehensive view of cellular interactions and microenvironments, elucidating mechanisms underlying islet function, survival, and rejection. This understanding is instrumental in developing targeted therapies to enhance graft performance and patient outcomes. The review emphasizes the significance of these research avenues in informing clinical practices and improving outcomes for patients with DM through more effective islet transplantation strategies. Future research directions include the application of these technologies in personalized medicine, developmental biology, and regenerative medicine, with the potential to predict disease progression and treatment responses. Addressing ethical and technical challenges will be crucial for the successful implementation of these integrated approaches in research and clinical practice, ultimately enhancing our ability to manage DM and improve patient quality of life.

## 1 Introduction

Diabetes mellitus (DM) is a burgeoning global health crisis, with the International Diabetes Federation (IDF) estimating that over 537 million adults are currently affected, a figure expected to grow to 643 million by 2030 (1). This increase, particularly in developing countries, exacerbates the strain on healthcare systems, necessitating innovative treatments like islet transplantation (2). Islet transplantation holds particular promise for DM management by restoring insulin production and metabolic control (3). However, the procedure faces challenges such as a lack of donor organs, lifelong immunosuppression requirements, and the risk of islet loss due to autoimmune or inflammatory responses (4). To overcome these, research into biomaterials and encapsulation techniques aims to improve patient outcomes (5, 6).

Recent advancements in single-cell RNA sequencing (scRNA-seq) and spatial transcriptomics (ST) are transforming our comprehension of complex biological processes, particularly in the context of islet transplantation and DM management (7, 8). ScRNA-seq allows for the analysis of gene expression patterns on a per-cell basis, shedding light on the heterogeneity of cell populations within islets and their responses to transplantation (8). This technology is crucial for identifying specific cell types, their states, and interactions within the graft microenvironment, which can inform strategies for enhancing transplant outcomes and improving patient management. ST, on the other hand, complements scRNA-seq by mapping gene expression within the spatial context of tissues. This is essential for understanding the microenvironment surrounding transplanted islets and their interactions with host tissues. The spatial information provided by this technology helps to reveal how different cell types and their functions are organized within the tissue, which is critical for the success of islet transplantation.

The integration of these technologies enables researchers to explore cellular heterogeneity and spatial organization simultaneously, leading to a more holistic view of cellular interactions and microenvironments. This integrative approach is crucial for elucidating complex biological processes and disease mechanisms, and recent studies have demonstrated the efficacy of computational tools, such as the iSpatial algorithm. This facilitates the analysis of high-dimensional datasets, paving the way for novel discoveries in tissue biology and pathology (9).

The integration of scRNA-seq and ST provides a comprehensive view of cellular interactions and microenvironments, crucial for understanding complex biological processes (9). Recent studies, including high-plex protein and transcriptome co-mapping with spatial CITE-seq (10), multimodal tri-omics mapping of brain development and neuroinflammation (11), and spatially resolved *in vivo* CRISPR screening via Perturb-DBiT (12), demonstrate the efficacy of these technologies in revealing cellular heterogeneity and spatial organization. These advancements offer new insights into gene function and regulation, enhancing our ability to develop targeted therapies for islet transplantation and DM management.

This review aims to provide an overview of research on single-cell and ST in islet transplantation for DM treatment. By integrating these cutting-edge technologies into the study of islet transplantation, researchers can enhance the efficacy of this promising therapeutic approach and improve outcomes for patients suffering from DM through more effective islet transplantation strategies.

## 2 ScRNA-seq in islet transplantation

ScRNA-seq has emerged as a transformative method in islet transplantation, offering unprecedented insights into cellular heterogeneity and functionality (13). This technology allows researchers to dissect the complex cellular composition of islet grafts, enhancing our understanding of graft acceptance, function, and failure. The application of scRNA-seq is pivotal for identifying specific cell types, their states, and interactions within the graft microenvironment, which can inform strategies for enhancing transplant outcomes and improving patient management (14).

### 2.1 Overview of scRNA-seq technology

ScRNA-seq has revolutionized the study of cellular heterogeneity by quantifying unique gene expression profiles for each cell, providing a detailed view of the transcriptomic landscape (12). Recent advancements have improved resolution and throughput, enabling the analysis of thousands of cells simultaneously and facilitating the identification of specific cell types and their activation states (15). This technology is particularly advantageous in islet transplantation, where understanding distinct cellular populations and their functional states is crucial. Genomic medicine’s ongoing evolution is set to enhance clinical applications and expand genomics’ role in practice (16, 17). Best practices for scRNA-seq have been compiled to offer a comprehensive guide for beginners and update advanced users on the latest methods (18).

### 2.2 ScRNA-seq insights into islet cell heterogeneity and DM

Recent advancements in scRNA-seq have greatly expanded our comprehension of islet cell dysfunction in DM (19, 20). Integration of chromatin accessibility, gene expression, and functional data with genetic associations has identified disease-causal gene regulatory changes, revealing two subtypes of β cell subtypes that shift in prevalence as type 2 DM progresses (21). A single-cell atlas of mouse islets (MIA) (22) and dynamic scRNA-seq of human pancreatic slices (HPSs) (23) have provided comprehensive resources for exploring pancreatic cell states and tissue plasticity. These studies have defined transcriptional signatures under endoplasmic reticulum and inflammatory stress, identified unique cell type responses, and highlighted the heterogeneity of T cell responses to insulin-derived epitopes (24). Additionally, human vascularized macrophage-islet organoids have modeled immune-mediated beta cell pyroptosis following viral infections, uncovering mechanisms of beta cell damage (25). Together, these discoveries highlight the complexity of immune responses and cellular heterogeneity in islet transplantation and DM, offering new avenues for targeted therapeutic interventions and improved treatment strategies.

### 2.3 Analysis of cellular heterogeneity in grafts

The application of scRNA-seq has unveiled significant cellular heterogeneity within islet grafts, which is crucial for understanding transplant outcomes. Islet cells are not homogeneous, consisting of various cell types, each playing distinct roles in glucose homeostasis (26). ScRNA-seq allows researchers to categorize these cells based on their transcriptional profiles, revealing subpopulations that may respond differently to transplant conditions. For instance, certain beta cell subtypes have been shown to exhibit greater resilience to stress and inflammation (27), which are key factors in islet graft survival. Furthermore, scRNA-seq enables the exploration of intercellular communication networks within the graft, highlighting how different cell types interact and influence each other’s functions. This understanding of cellular heterogeneity is essential for enhancing graft function and longevity, ultimately improving the management of DM in transplant recipients. Recent scRNA-seq studies have shed new light on the immune context of islet transplants, identifying T lymphocytes and myeloid cells as key post-transplant immune elements, particularly in allografts (8, 13). Significantly, allogeneic islet cells can adopt antigen-presenting cell-like properties, potentially enhancing interactions with CD8+ T cells and contributing to allograft destruction (7). These findings underscore the immune response in islet transplantation and the potential for targeted immunomodulatory strategies to enhance graft survival.

### 2.4 Impact of scRNA-seq on islet function recovery

ScRNA-seq has profound implications for the recovery of islet function following transplantation. By elucidating the cellular mechanisms that govern islet viability and function, researchers can identify potential therapeutic targets to enhance graft performance. For example, single-cell analyses have demonstrated that specific signaling pathways and gene expression profiles are associated with successful islet engraftment and function restoration (13). Moreover, understanding the dynamic changes in the islet cellular landscape post-transplantation can inform strategies to mitigate the effects of inflammation and immune rejection, which are significant barriers to long-term graft survival (28). The interventions aimed at modulating the immune response or enhancing the regenerative capacity of beta cells can lead to improved outcomes for transplant recipients (29). Thus, the insights gained from scRNA-seq are instrumental in advancing therapeutic strategies that promote islet recovery, ultimately contributing to better glycemic control and quality of life for patients with type 1 DM.

## 3 Research progress in ST

ST is a rapidly evolving field that integrates transcriptomic data with spatial information from tissue samples, allowing researchers to explore the complex cellular architecture of tissues in their native environments (30). This innovative technology overcomes the limitations of traditional scRNA-seq, which lacks spatial context (31). ST provides insights into cellular interactions and the microenvironment, which are crucial for understanding various biological processes and disease mechanisms. Recent advancements in ST techniques, such as multiplexed barcoding and high-resolution imaging, have significantly improved the ability to analyze large tissue sections, enabling the study of heterogeneous tissues, including tumors and organs affected by various diseases (32). The continuous development of ST provides a more comprehensive understanding of tissue biology.

### 3.1 Introduction to ST technology

ST technology, which enables the direct mapping of gene expression profiles onto tissue sections, has revolutionized our ability to visualize the spatial distribution of RNA molecules, thereby revealing the complex cellular organization and interactions within tissues (33). This approach has been further enhanced by the integration with scRNA-seq, allowing for the identification of distinct cell populations and their functional states within the tissue microenvironment (33). The technology has shown tremendous potential in disease understanding and drug discovery, providing new perspectives for drug target identification and pharmacological research model building (34). Moreover, spatially resolved transcriptomic technologies have accelerated discovery in fields, offering insights into cell-cell interactions and changes in response to injury (35). As the field progresses, the challenges and opportunities in translating high-resolution ST into clinical practice are being addressed, with the potential to identify disease mechanisms and guide personalized therapies (36). Overall, ST stands as a pivotal tool in advancing our understanding of tissue biology and driving the development of precision medicine.

### 3.2 Spatial construction and dynamic monitoring of the islet microenvironment

The pancreatic islet microenvironment is critical for preserving the function and survival of islet cells in DM and islet transplantation (37). Recent studies have utilized ST to construct a detailed spatial map of the islet microenvironment, highlighting the interactions between different cell types (38). This spatial analysis reveals how the local cellular context influences islet function and response to metabolic challenges. Furthermore, dynamic monitoring of the islet microenvironment using ST allows researchers to observe shifts in gene expression profiles over time, yielding insights into the causes of islet dysfunction and the effects of therapeutic interventions. For instance, studies have shown that alterations in the spatial arrangement of immune cells in the islet can contribute to autoimmune attacks in T1D (39). By elucidating the spatial dynamics of the islet microenvironment, ST holds promise for improving strategies in islet transplantation and developing targeted therapies for DM.

### 3.3 Analysis of the impact of ST on the graft microenvironment

ST holds the potential to significantly advance our understanding of the graft microenvironment in transplantation biology in the future. This technology, when applied to islet transplantation, could offer a spatially detailed perspective on gene expression in graft tissues, potentially enabling the detection of molecular patterns that correlate with successful graft integration and function. While current research has not yet fully explored this area, the technology’s promise suggests it could reveal the heterogeneity of immune cell populations surrounding the graft, which is crucial for understanding immune responses and potential rejection mechanisms. For instance, understanding the spatial distribution of T cells and regulatory cells within the graft could influence the outcome of transplantation. Additionally, ST could facilitate the exploration of interactions between graft cells and the surrounding microenvironment, including the extracellular matrix and signaling molecules. This comprehensive analysis could inform the development of novel immunomodulatory strategies aimed at enhancing graft survival and function. As the field progresses, integrating ST with other omics technologies may further enhance our ability to dissect the complex biological processes governing graft success and failure.

## 4 The integration of scRNA-seq and ST

Recent advancements in scRNA-seq and ST offer exciting new avenues for understanding the complexities of islet transplantation and DM management. ScRNA-seq enables cell-level gene expression analysis, revealing cell populations within islets and their response to transplantation. ST, on the other hand, enables the mapping of gene expression within tissues, which is crucial for understanding the microenvironment surrounding transplanted islets and their interactions with host tissues. These technologies hold immense potential for elucidating the mechanisms underlying islet function, survival, and rejection, opening up new avenues for improved therapeutic strategies in DM management. Combining single-cell multi-omics with existing knowledge allows for functional insights into gene regulation and cell interactions, which is particularly relevant in immunology (40). ST has become a key instrument in disease understanding and drug discovery, offering new perspectives into how drugs interact with targets and their mechanisms of action (34). ScRNA-seq and spatial multi-omics is rapidly evolving, with methods such as ST playing a significant role in uncovering spatial heterogeneity and creating comprehensive spatial maps (41), which is particularly relevant in the context of islet transplantation and DM management. The application of spatial multi-omics in unraveling the synergy between different omics data highlights the importance of these approaches in advancing our understanding of complex diseases and their treatments (42). Furthermore, the potential of single-cell and spatial resolution in deciphering drug targets and actions cannot be overlooked (43), which is crucial for the development of personalized therapeutic strategies in DM.

### 4.1 Future research directions and clinical application prospects

The future of research in the integration of scRNA-seq and ST is promising, with several potential directions for exploration. One significant area of interest is the application of these technologies in personalized medicine, where understanding the unique cellular composition and gene expression profiles of individual patients can lead to tailored therapeutic strategies (34, 44). Furthermore, the integration of single-cell and spatial transcriptomic data with clinical outcomes could enhance our ability to predict disease progression and treatment responses (45).

### 4.2 Computational tools and frameworks

To fully harness the potential of scRNA-seq and ST, the development of integrative analysis methods is essential (46). These methods address the challenges associated with the high dimensionality and complexity of the data generated by these technologies. Various computational frameworks have been proposed to facilitate the synthesis of multi-omics data, allowing researchers to analyze gene expression, protein levels, and epigenetic modifications concurrently (47). For instance, recent advancements in machine learning and statistical modeling have enabled the concurrent analysis of scRNA-seq and spatial transcriptomic data, providing insights into the interplay between cellular states and their spatial contexts (48). Additionally, the application of integrative methods, such as multi-dimensional scaling and clustering algorithms, aids in the identification of distinct cell populations and their spatial distributions within tissues (49). The computational tools used for integrating scRNA-seq and ST data published in high-impact journals after 2023 were summarized in **Table 1**. These advancements and tools highlight the progress in multi-modal single-cell omics and computational integration, providing researchers with a more comprehensive and robust framework for analyzing complex biological systems. By incorporating these cutting-edge methodologies, the field of islet transplantation and DM management can benefit from a deeper understanding of cellular heterogeneity and spatial organization, leading to more effective therapeutic strategies.

**Table 1:** Computational tools for integrating single-cell and spatial transcriptomics data.

Computational Tools	Strategy Type	Ref
SPASCER	ST annotation at single-cell resolution, database	(50)
MARIO	Robust single-cell matching and multimodal analysis	(51)
SPICEMIX	Integrative single-cell spatial modeling of cell identity	(52)
PRECAST	Probabilistic embedding, clustering, and alignment for integrating ST data	(53)
GraphST	Graph self-supervised contrastive learning	(54)
spSeudoMap	Cell type mapping of ST using unmatched single-cell RNA-seq data	(55)
scSpace	Reconstruction of the cell pseudo-space	(56)
UnitedNet	Multi-modal biological data analysis	(57)
BulkSignalR	Inferring ligand-receptor cellular networks	(58)
CAJAL	Analysis and integration of single-cell morphological data	(59)
MaxFuse	Integration of spatial and single-cell data	(60)
SiGra	Single-cell spatial elucidation through an image-augmented graph transformer	(61)
SpatialScope	Deep generative model	(46)
Smoother	Unified and modular framework for incorporating structural dependency in spatial omics data	(62)
SpaTrio	Revealing spatial multimodal heterogeneity	(63)
iStar	Integration of ST with histology	(64)
SLIDE	Significant latent factor interaction discovery	(65)
SpaDo	Multi-slice spatial transcriptome domain analysis	(66)
DANCE	Deep learning library and benchmark platform for single-cell analysis	(67)
Starfysh	Integrates spatial transcriptomic and histologic data to reveal heterogeneous tumor-immune hubs	(68)
Bento	Subgraph-based graph attention network	(69)
DOT	Flexible multi-objective optimization framework for transferring features across single-cell and spatial omics	(70)
ezSingleCell	Integrated one-stop single-cell and spatial omics analysis platform for bench scientists	(71)
Panpipes	Pipeline for multiomic single-cell and spatial transcriptomic data analysis	(72)
aKNNO	Single-cell and ST clustering with an optimized adaptive k-nearest neighbor graph	(73)
STdGCN	Spatial transcriptomic cell-type deconvolution using graph convolutional networks	(74)
StaVia	Spatially and temporally aware cartography with higher-order random walks for cell atlases	(75)
DeepTalk	Subgraph-based graph attention network	(76)
METI	Deep profiling of tumor ecosystems by integrating cell morphology and ST	(77)
scConfluence	Single-cell diagonal integration with regularized Inverse Optimal Transport on weakly connected features	(78)
CAST	Single-cell resolution search and match	(79)
SpaTopic	Statistical learning framework for exploring tumor spatial architecture from spatially resolved transcriptomic data	(80)
Vitessce	Integrative visualization of multimodal and spatially resolved single-cell data	(81)
PANDA	Dual decoding of cell types and gene expression	(82)
scDOT	Optimal transport for mapping senescent cells in ST	(83)
scProAtlas	Atlas of multiplexed single-cell spatial proteomics imaging in human tissues	(84)

### 4.3 Clinical implementation challenges and solutions

While the potential clinical applications of scRNA-seq and ST are significant, several barriers must be addressed to facilitate their widespread use in clinical settings. Cost is a major factor, with the high expense of these technologies currently limiting their use in routine clinical diagnostics. However, as technology advances and the market expand, costs are expected to decrease, making these tools more accessible. The need for specialized equipment also poses a challenge for many medical institutions. Research is ongoing to develop more convenient and cost-effective devices, reducing the dependence on high-end equipment. Additionally, handling large numbers of samples is a current limitation. Optimizing experimental processes and data analysis methods, as well as improvements in computational algorithms and data management systems, can enhance the scalability of these technologies, making them more feasible for clinical use.

### 4.4 Ethical challenges

Despite the exciting prospects of integrating scRNA-seq and ST, several ethical challenges must be addressed. Ethically, the collection and use of biological samples raise significant concerns regarding informed consent, privacy, and data security, particularly when dealing with sensitive patient information. The potential for misuse of genetic data necessitates robust ethical guidelines and regulatory frameworks to protect individuals’ rights. Ensuring that patients are fully informed about the purposes, risks, and benefits of the samples being used in research is crucial. Additionally, maintaining the confidentiality and security of genetic data is essential to prevent unauthorized access and potential discrimination. Addressing these ethical challenges will be crucial for the successful implementation of integrated single-cell and spatial transcriptomic approaches in both research and clinical practice.

## 5 Integration into clinical workflows

Pre-transplant, scRNA-seq can identify optimal donor-recipient matches by analyzing islet cell subsets, as demonstrated in highly purified human islet cell studies. ST can monitor disease progression, aiding in timing transplant decisions. Post-transplant, scRNA-seq can detect early signs of graft rejection, while ST assesses graft integration. Collaboratively, bioinformaticians and clinicians can use tools like SpatialScope to integrate these data, enhancing our understanding of the transplant microenvironment. Standardization and benchmarking, as well as advancements in permeabilization and single-cell resolution technologies, are crucial for improving these methods. These integrations and collaborations will lead to more effective therapeutic strategies and improved patient outcomes.

## 6 Limitations of current methods

### 6.1 Limited spatial resolution and cell-to-cell interaction analysis

ScRNA-seq provides high-resolution identification of cell types and their states but lacks the ability to pinpoint their spatial distribution or capture local cell–cell interactions and the ligands and receptors that mediate these interactions. This limitation hinders our ability to fully explore the islet microenvironment and the complexity of cell–cell interactions therein. ST, while providing spatial context, often has limitations in spatial resolution. For example, the Visium platform, despite its widespread use, has a spot size of 55 μm, which often contains multiple cells, limiting its ability to resolve detailed tissue structure and characterize cellular communications.

### 6.2 Systematic benchmarking and standardization

While imaging-based ST has a longer history and a collaborative benchmarking effort has been initiated with the SpaceTX consortium, a systematic benchmarking study has not been done for ST. This lack of benchmarking complicates the establishment of universal evaluation standards. Moreover, ST technologies diverge notably in aspects such as spatial resolution and the preparation of spatially barcoded oligo arrays. This variability introduces challenges in method selection and complicates the establishment of universal evaluation standards.

### 6.3 Gene detection biases and sensitivity

Unexpected gene-capturing biases were existed in the polyA-based Visium platform, with marker genes consistently captured by other technologies not showing up in the Visium (polyA) data. Considering Visium is the most widely used commercial platform, it is important to further verify its gene-capturing bias on other tissues. Besides, the spot size has become an important metric as a surrogate of the resolution for each method. However, diffusion is a key factor that affects the actual resolution. Varying the permeabilization time has a substantial impact on diffusion, and different technologies exhibited varied diffusion profiles across various tissue types.

### 6.4 Future directions to address these limitations

To address the limitations of current methods in integrating scRNA-seq and ST data, we propose a multi-faceted approach. First, we suggest using deep generative models like SpatialScope to integrate scRNA-seq and ST data, achieving single-cell resolution and a comprehensive understanding of the transcriptome’s spatial distribution. Additionally, developing accurate statistical and computational methods, such as multi-dimensional scaling and clustering algorithms, is crucial for handling the data’s complexity. Second, we recommend establishing a standardized benchmarking framework and generating cross-platform datasets to evaluate ST methods systematically. Finally, optimizing permeabilization and developing technologies for true single-cell resolution, such as Slide-tag, will improve sensitivity and provide more detailed spatial information. These advancements will enhance the utility of scRNA-seq and ST in islet transplantation and DM management, leading to more effective therapeutic strategies.

## 7 Conclusion

**Figure 1:**
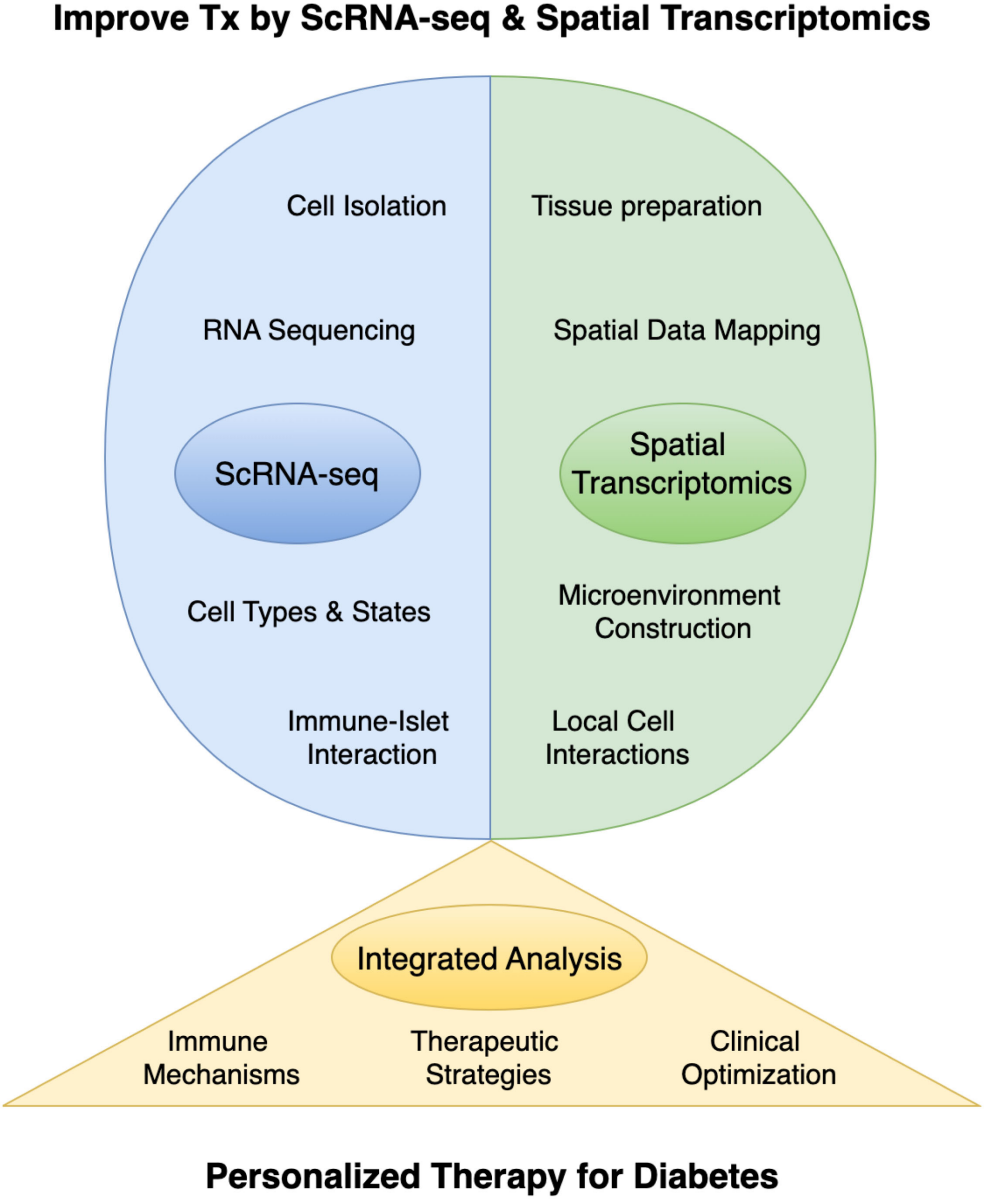
The application of single-cell RNA sequencing (scRNA-seq) and spatial transcriptomics in islet transplantation research. Tx, Islet Transplantation.

Single-cell omics and ST have revolutionized islet transplantation research, providing detailed insights into cellular diversity and spatial organization (**Figure 1**). These technologies not only reveal the complex interactions within the transplant microenvironment but also identify key regulatory pathways and therapeutic targets to enhance graft survival and function. The integration of these approaches has significant translational potential, offering a more comprehensive framework for improving clinical outcomes in patients with DM. Future research should focus on longitudinal studies and interdisciplinary collaborations to fully realize this potential, ensuring that emerging discoveries translate effectively into clinical practice.
